# The human intervertebral disc as a source of DNA for molecular identification

**DOI:** 10.1007/s12024-021-00401-0

**Published:** 2021-07-22

**Authors:** Julia Becker, Nina Sophia Mahlke, Stefanie Ritz-Timme, Petra Boehme

**Affiliations:** Institute of Legal Medicine, University Hospital Düsseldorf, Moorenstraße 5, 40225 Düsseldorf, Germany

**Keywords:** Short tandem repeats, Molecular identification, Intervertebral disc, Decomposition

## Abstract

Genetic analyses such as STR-typing are routinely used for identification purposes in forensic casework. Although genotyping techniques only require a minimum amount of DNA to provide a genetic profile, DNA quality differs not only between but also within tissues during ongoing decomposition. Initiated by a recent case where, due to the constitution of the body, preferred tissue was not available or only resulted in a partial and not usable DNA profile, the analysis of intervertebral discs as a source of DNA was considered. As the analysis of this tissue resulted in a high quality DNA profile a further study was performed in which thirty intervertebral discs dissected from bodies in different stages of decay were analyzed. All samples yielded good quality DNA in quantities suitable for STR-based amplification with no or only low degradation indices, resulting in complete genetic profiles. These results demonstrate the robustness of human intervertebral disc tissue as a source of DNA for molecular identification purposes.

## Introduction

STR-based DNA profiling has become the gold standard for identification purposes, especially for highly decomposed bodies or body fragments [1–3]. Fortunately, all human tissue contains cell nuclei and very small amounts can be used for DNA extraction [4–9]. However, high quality DNA is typically restricted to fresh tissue samples. Decomposition processes and/or environmental factors reduce the stability and integrity of DNA within and between tissues [10–12]. Thus, for bodies in advanced stages of putrefaction, when suitable samples of organs and muscles may no be longer available, solid material such as teeth or bones have to be used for analysis [13, 14]. However, processing may be time-consuming and/or technically difficult and may depend on experience [15, 16]. Cartilages and ligaments, like the Achilles tendon, are quite resistant to autolysis and putrefaction and thus well suited for molecular identification even in heavily decomposed bodies [17–21]. When preferred tissue is not available or is too degraded to generate complete genetic profiles, the analysis of intervertebral discs (IVDs) may be considered. IVDs are composed of two main components, namely the annulus fibrosus that consists of concentric fibrous layers and the gelatinous centralized nucleus pulposus. In general, the extracellular matrix of the IVD is comprised of collagen, elastin and other non-collagenous components [22]. The cells are embedded in the matrix and due to the absence of blood vessels, the matrix may serve as a physical barrier that reduces the tissue's susceptibility to degradation processes. The present study does not intend to demonstrate that IVDs are more suitable than other material but to show that they can be considered as a good source of DNA for molecular identification.

## Material and methods

### Sample collection

Intervertebral discs were collected from 30 bodies (23 males, 7 females; 24 to 87 years old) during autopsy at the Institute of Forensic Medicine Düsseldorf, Germany (Table 1). Twenty-three bodies were in different stages of decay (fresh to advanced/skeletonized; defined by forensic pathologists based on morphological characteristics as described by Megyesi et al. [23]) and seven bodies were either exhumed, burnt or drowned. IVDs were dissected between the lumbar vertebral bodies 2 and 3 of the spinal column. One body, however, was in such an advanced stage of decomposition that due to partial skeletonization and exposed limbs only the IVD between the lumbar vertebral bodies 4 and 5 could be collected. The postmortem interval, if known, ranged between 1 to 28 days. All tissue samples were stored at -80 °C until further processing.

**Table 1:** Samples from bodies in different stages of decay. State of decay according to Megyesi [23] for the entire body, appearance of the IVDs postmortem intervals (as far as known), DNA concentration (in ng/µl) and degradation indices (both with standard deviation) measured in duplicates for each sample. m = male, f = female, age in years. Asterisk (*) = bodies with a partially skeletonized torso; black circle (•) = body where only IVD between the lumbar vertebral bodies 4 and 5 could be collected; A-D = STR profile (blue dye channel) shown in Fig. 1

Age and sex	Stage of decomposition	Appearance of intervertebral discs	Postmortem interval	DNA concentration	Degradation index
36, m ^A^	Fresh	No abnormalities	< 3 days	34.02 ± 0.92	1.11 ± 0.02
68, m	Fresh	No abnormalities	< 3 days	32.23 ± 1.16	1.15 ± 0.05
50, m	Fresh	No abnormalities	< 3 days	14.42 ± 0.52	1.13 ± 0.01
87, f	Fresh	No abnormalities	< 3 days	39.71 ± 0.79	2.62 ± 0.06
28, m	Fresh	No abnormalities	< 3 days	5.28 ± 0.44	1.68 ± 0.03
46, m	Fresh	No abnormalities	< 3 days	13.89 ± 0.02	1.24 ± 0.05
70, f	Fresh	No abnormalities	< 3 days	4.64 ± 0.18	1.06 ± 0.02
			**Mean**	**20.74 ± 13.87**	**1.42 ± 0.54**
39, m	Early	No abnormalities	unknown	8.40 ± 1.09	1.20 ± 0.01
24, m	Early	No abnormalities	unknown	19.63 ± 0.00	1.41 ± 0.02
28, m	Early	No abnormalities	unknown	14.93 ± 2.50	1.38 ± 0.02
64, m	Early	No abnormalities	< 14 days	24.24 ± 0.00	2.80 ± 0.07
47, m	Early	No abnormalities	approx. 4 days	3.67 ± 0.42	1.39 ± 0.02
70, f	Early	Brown discoloration	< 7 days	20.03 ± 0.00	1.63 ± 0.04
65, m	Early	Brown discoloration	< 10 days	11.45 ± 1.96	1.37 ± 0.01
52, m	Early	Brown discoloration	unknown	1.51 ± 0.09	1.29 ± 0.06
61, f	Early	Green discoloration	unknown	5.50 ± 0.52	4.46 ± 0.26
61, m	Early	Green discoloration	unknown	13.10 ± 0.05	1.29 ± 0.02
			**Mean**	**13.19 ± 7.12**	**1.59 ± 0.48**
60, m	Advanced	No abnormalities	unknown	3.74 ± 0.26	1.64 ± 0.07
31, m	Advanced	Brown discoloration	unknown	12.00 ± 0.04	1.45 ± 0.09
66, f	Advanced	Brown discoloration	unknown	4,57 ± 0.18	1.47 ± 0.02
45, m	Advanced*	Brown discoloration	unknown	4.04 ± 0.14	1.50 ± 0.02
47, m	Advanced	Brown discoloration	< 21 days	16.05 ± 0.83	2.02 ± 0.13
65, m•^B^	Advanced*	Brown discoloration	21–28 days	11.41 ± 0.98	1.92 ± 0.03
			**Mean**	**7.06 ± 3.93**	**2.05 ± 1.15**
	**Others**				
79, m	Exhumed	No abnormalities	approx. 2 months	70.20 ± 0.00	1.67 ± 0.07
83, m	Exhumed	No abnormalities	approx. 2 months	13.51 ± 0.05	1.52 ± 0.05
84, m	Exhumed	No abnormalities	approx. 2 months	5.31 ± 0.58	1.48 ± 0.04
48, m ^D^	Severe burns	No abnormalities	< 1 day	15.11 ± 0.30	1.41 ± 0.03
58, f	Severe burns	No abnormalities	< 1 day	14.12 ± 0.21	1.12 ± 0.00
58, m	Most severe burns	No abnormalities	< 1 day	3.13 ± 0.24	1.11 ± 0.01
57, f ^C^	Drowned	No abnormalities	approx. 1 month	11.40 ± 0.00	1.53 ± 0.04

### DNA extraction

Prior to DNA extraction, ligament residues were removed from the IVDs using a sterile scalpel. Subsequently, an approximately 3 × 2 mm piece was cut out of the outer layers of the anterior annulus fibrosus for DNA extraction using a silica-based method (NucleoSpin® Tissue Kit from Macherey–Nagel, Düren, Germany). The extraction was performed according to the manufacturer's instruction with overnight at 56 °C in a shaking thermal block (ThermoMixer® C, Eppendorf). DNA was eluted in 50 µl BE buffer (included in Macherey–Nagel kit). DNA extracts were stored at -20 °C.

### Quantification and amplification

Quantitation was performed in duplicates using the Applied Biosystems™ 7500 Real-Time PCR System and the Quantiplex® Pro Kit (Qiagen) following manufacturer’s instructions with default settings. A multiplex PCR for 17 STR loci (PowerPlex® ESI 17 Fast System, Promega) was performed in a total reaction volume of 12.5 µl. Where possible an optimum of 0.5 ng template DNA was added to the PCR reaction. Thermal cycling conditions were followed as described by the manufacturer. Capillary electrophoretic separation was performed on the ABI Prism® Genetic Analyzer 3130 equipped with a 36 cm Capillary Array/POP-4 (Applied Biosystems, Darmstadt, Germany) following manufacturer’s instructions. Data acquisition and analysis was performed using the ABI Prism 3130 Collection software (Applied Biosystems, Darmstadt, Germany) and GeneMapperID® v.3.2 software (Applied Biosystems, Darmstadt, Germany).

## Results

### Morphological features of the intervertebral discs

Because a standardized removal of IVDs turned out to be challenging due to degenerative changes and/or the positioning of the spine, the appearance of the dissected IVDs differed. In most cases, it was possible to dissect the complete IVD, in some cases only the anterior part or even fragments could be removed. Ten intervertebral discs of bodies that were in an advanced stage of putrefaction showed slight discolorations (green to brown) (Table 1). The remaining samples showed no noteworthy discolorations.

### Quantification analysis

Quantitation yielded sufficient amounts of DNA for further STR-analysis in all samples (Table 1). Quantity, however, was very variable between samples and ranged from 1.51 to 70.2 ng/µl with standard deviations between 0.02 to 2.50 ng/µl. The amount of extracted DNA was significantly higher for IVDs collected from fresh bodies (mean value: 20.60 ng/µl ± 14.45 ng/µl) than for IVDs from bodies in an initial to advanced stage of decay (10.89 ng/µl ± 6.84 ng/µl) with a p-value of 0.0165 (two tailed Welch t-test with α = 0.05). Detailed information about quantity and quality is summarized in Table 1 for each stage of decomposition.

### DNA profile quality

**Fig. 1:**
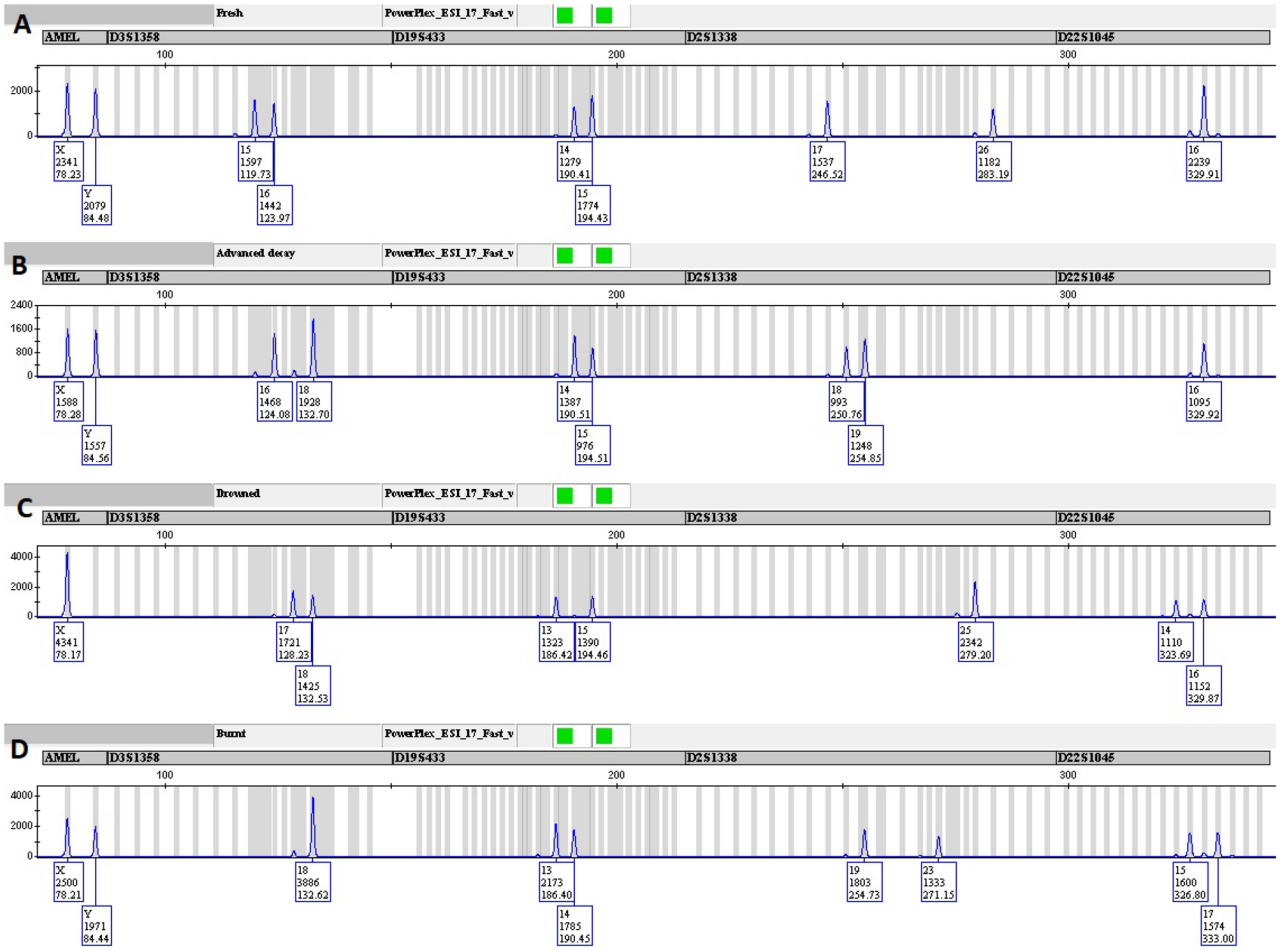
DNA profiles (blue dye channel, PowerPlex® ESI 17 Fast System) of bodies in the following stages of decomposition: **A**) fresh **B**) advanced **C**) burnt **D**) drowned. The corresponding bodies are marked in Table 1

Although variation in quantity was observed, there was no measurable inhibition or noteworthy degradation (degradation indices between 1.06 and 4.46; kit-specific threshold with a default setting index of 10) in any of the 30 IVD samples and STR-analysis resulted in full and single-source DNA profiles with no artefacts (Fig. 1).

## Discussion

The inverse relationship between the degree of decomposition and quantity of extracted DNA may be explained by an increasing DNA degradation in terms of fragmentation with increasing degree of putrefaction [24–26]. This relationship was also observed in an in vitro study in which IVDs putrefied under controlled conditions (dry and wet) for several days to simulate longer postmortem intervals (data not shown). Interestingly, neovascularization, i.e. the formation of new blood vessels, may occur in IVDs [27, 28] and thus, decay-induced microorganisms may have easier access, which may accelerate the breakdown of tissue. A variation between samples (within one stage of decay) may be due to the distribution of cells (e.g. clusters, single cells) [29, 30], a varying thickness (i.e. height) of the removed IVDs, or differences in bacterial colonization [31]. However, morphological appearance, i.e. discoloration is not related to quantity and/or quality of extracted DNA. According to our data, human intervertebral disc tissue is very resistant against degradation processes and may be considered as source of DNA for STR-based identification.
